# Empirical Study on Classifiers for Earlier Prediction of COVID-19 Infection Cure and Death Rate in the Indian States

**DOI:** 10.3390/healthcare10010085

**Published:** 2022-01-02

**Authors:** Pratiyush Guleria, Shakeel Ahmed, Abdulaziz Alhumam, Parvathaneni Naga Srinivasu

**Affiliations:** 1 National Institute of Electronics and Information Technology, Shimla 171001, India; pratiyushguleria@gmail.com; 2 Department of Computer Science, College of Computer Sciences and Information Technology, King Faisal University, Al-Ahsa 31982, Saudi Arabia; aahumam@kfu.edu.sa; 3 Department of Computer Science and Engineering, Gitam Institute of Technology, GITAM Deemed to be University, Visakhapatnam 530045, India; parvathanenins@gmail.com

**Keywords:** classifier, fine-tuned ensemble classification, disease prediction, decision making, forecasting, machine learning models

## Abstract

Machine Learning methods can play a key role in predicting the spread of respiratory infection with the help of predictive analytics. Machine Learning techniques help mine data to better estimate and predict the COVID-19 infection status. A Fine-tuned Ensemble Classification approach for predicting the death and cure rates of patients from infection using Machine Learning techniques has been proposed for different states of India. The proposed classification model is applied to the recent COVID-19 dataset for India, and a performance evaluation of various state-of-the-art classifiers to the proposed model is performed. The classifiers forecasted the patients’ infection status in different regions to better plan resources and response care systems. The appropriate classification of the output class based on the extracted input features is essential to achieve accurate results of classifiers. The experimental outcome exhibits that the proposed Hybrid Model reached a maximum F1-score of 94% compared to Ensembles and other classifiers like Support Vector Machine, Decision Trees, and Gaussian Naïve Bayes on a dataset of 5004 instances through 10-fold cross-validation for predicting the right class. The feasibility of automated prediction for COVID-19 infection cure and death rates in the Indian states was demonstrated.

## 1. Introduction

The COVID-19 respiratory disease is the name for the Coronavirus and is a type of SARS-CoV-2 virus. This respiratory infectious virus replicates from human to human. The symptoms of the diseases are related to the flu and common cold disorders, but the Coronavirus attacks the respiratory system of a human being. This Coronavirus outbreak has resulted in crises across the globe. COVID-19 has affected human life on a large scale and primary services like travel, supply chain, essential daily needs, etc. The best efforts are being put forth globally to control this pandemic situation. To stop the spread of the virus, researchers, scientists, health organizations, pharmaceutical companies, and many frontiers across different countries are working together. There are collaborative efforts from many research institutions in areas like discovering the antiviral against valid viral targets, diagnostics for symptomatic and asymptomatic respiratory viral infections, finding vaccines against the respiratory virus, developing diseases paradigm for respiratory viral infections, finding natural methods to increase immunity to fight against the virus, and the epidemiological study of COVID-19 and other common respiratory viral infections. The health organizations, scientists, administration, and government bodies of different nations have discovered medicines and vaccines to control the disease. In this context, Machine Learning (ML) and Artificial Intelligence (AI)-enabled technology initiatives have also contributed to the medical science curtailing the pandemic [1].

Machine Learning improvises through self-learning from experiences without explicitly programming the Machine Intelligence models [2]. The Data Science and ML technologies have come into force for predicting the following: (a) the outbreak of the virus, (b) diagnosing patients based on the symptoms, (c) analyzing the severity of the virus on specific age groups, (d) the severity of the virus on patients with other health issues, (e) finding a cure from virus and drug discovery, (f) analyzing patients who recovered from the virus, (g) study environment suitability for the sustainability of the virus, (h) interpreting the possibility of virus activation in the coming years, (i) unlocking the coronavirus vaccine, and (j) analyzing and predicting the structure of the protein of COVID-19.

The classification algorithms in Data Mining use Natural Language Processing (NLP) and Machine Intelligence techniques to extract meaningful information from divergent sources to identify the early signs of an infectious outbreak [3]. The basis for the collection of data involves (a) hospitals, (b) health centers, (c) railway, (d) airlines, (e) weather reports, (f) medical reports, etc. Now, AI algorithms assist humans in providing pertinent information related to COVID-19 obtained from all these sources on a day-to-day basis. The data find interesting patterns, like passenger traveling areas infected mostly from viruses and new locations. This specialization is also required from the fields of (a) GIS, (b) geographical data analytics, (c) building data visualization, (d) experts from the computer and medical sciences, (e) telemedicine, (f) the pharma sector, community health, etc. 

The Artificial Intelligence techniques help track infectious diseases, forecast the early signs of an outbreak, and speculate the peak level of virus spread [4]. The BlueDot, an AI platform, is tracking diseases in China by predicting the contamination of viruses based on the travel pattern of the people from one city to another [5]. Companies like Deep Mind are working on AI-based systems, and deep learning strategies are implemented for the detection and analysis of chest infections and body temperatures due to COVID-19 [6]. Artificial Intelligence methods like the Expert Systems (ES) may assist in offering quick expert advice based on their knowledge base [7]. The Data Mining techniques are crucial in ML for classifying and clustering data. The Data Mining techniques for data classification are Decision Trees, Regression, SVM, K-Nearest Neighbors, Random forest, Association Rule Mining, etc. In contrast, data clustering is performed using K-Means and neural networks [8]. Apart from them, Machine Intelligence techniques perform predictive analytics related to respiratory infection. Machine Intelligence techniques are being used for training deep learning-based models for personalized healthcare to patients at the preliminary stages. The trained models are helpful to doctors for the discovery of effective viral therapies and the identification of the common symptom clusters.

Early assessment of a COVID-19 outbreak allows for the rapid and effective treatment of COVID-19 and reduces healthcare costs that have motivated the current study on the impact analysis. An attempt has been made to aid healthcare practitioners around the globe in their triage of patients, particularly when faced with limited healthcare facilities, by developing prediction models that incorporate numerous indicators to assess the risk of infection. The prediction performance using Machine Learning models act as the valuable assistive tools for early clinical decision-making and resource allocation to prevent an increase in the mortality rate due to a lack of preparedness of proper medical infrastructure.

In the current study, various state-of-the-art classifiers for predicting the pandemic infection status in different states have done a performance evaluation of the classifiers in predicting the death and the cured rates of patients in different states. The predictive results of the death and cured rates of different classifiers are compared to the proposed hybrid classifier. The results obtained help in the pattern analysis of the infection affecting people and the cured rate from infection on a day-to-day basis in different states of India. The motivation behind this work is to alarm the local authorities to increase the readiness for efficient resource allocation from the results of the proposed work. Early predictions of mortality cases using classification techniques give insight into the local authorities to prepare with immediate medical assistance to the patients and efficiently manage the medical resources well in advance.

Following the introduction of the ensembled models, the literature review is discussed in Section 2, followed by Section 3, which presents various existing Ensemble Classifications and their performances. Section 4 presents the methodology adopted for the proposed model, and the details of the dataset and implementation environment are discussed. The results are discussed in Section 5, and the study is concluded in Section 6, followed by the future scope.

## 2. Literature Review

Healthcare hygiene is one of the emerging areas of Machine Intelligence technologies. ML focuses on the methods and tools for finding patterns in data. These patterns help in making predictions of risk factors of infections. Once trained from the patterns and outcomes, ML algorithms predict the test data [9]. Classification approaches are helpful in the healthcare sector, like in cases of heart diseases. An approach for classifying ECG signals is to use ML-based decision trees and random forest algorithms [10]. Alzubi et al. [11] reviewed the ML paradigms, learning processes, and scope of the models. Machine Learning-enabled technology is critical to extracting information from the data of many existing area scenarios. When COVID-19, a respiratory infectious disease caused by a new virus known as the Novel Coronavirus, Machine Learning algorithms came together to fight the virus outbreak, performing predictive analytics. The medicinal findings of COVID-19 are associated with acute respiratory distress syndrome. The findings show that Lymphopenia is a common feature in patients suffering from COVID-19 [12].

In most cases, the patients are cured, but it is possible only with social distancing and other preventive measures. Social distancing is the most critical factor to remain safe from this virus, as the virus spreads from human to human. This test, often referred to as RT-PCR, identifies COVID-19 in patient swab samples. Nasopharyngeal swabs are gathered to conduct the RT-PCR test. RNA, the genetic material of the virus, is extracted for analysis. The patient is diagnosed as affirmative if the genetic sequence of the SARS-CoV-2 virus is identical to theirs. An exploratory data analysis and visualization have been performed for virus-infected, recovered, and death cases through ML techniques [13]. The python programming language has algorithms for data classification, clustering, etc. for performing predictions [14]. A cohort study with Brazilian data was conducted by authors, and the outcome of the disease in COVID-19-positive patients through Machine Learning was predicted [15].

Big data and Machine Learning jointly work on applications of the healthcare industry. The challenge of the healthcare industry is to secure the electronic health record and its maintenance. The security issues of ensuring the medical industry data can be handled using Machine Learning and big data techniques [16]. The tools used to handle big data are Cassandra, Pig, Hive, HBase, HDFS, Zookeeper, etc. To control the outbreak of the virus, it is required to perform an accurate prediction of travel history; ML techniques need to be implemented [17]. In Machine Learning, programs learn from experiences and generalization. The classification algorithms perform iterations on the test dataset, which improves its performance automatically. The models are trained on the test dataset with no labels, classification, or categorization. The model derives from past experiences and performs on the unseen data. ML algorithms derive valuable patterns from the dataset [18]. The study conducted by students used Machine Learning for the analysis of COVID-19 and extracted public health insights [19]. Supervised ML techniques can be implemented with digital signal processing for a genome analysis. With Machine Learning, the classification of COVID-19 genomes was performed with better precision [20]. Rao et al. [21] proposed the finding of the Coronavirus more quickly through an AI framework. A mobile phone-based survey was conducted in provinces that are under quarantine. 

In the response to COVID-19 in Taiwan with the help of big data analytics, Taiwan is trying to resolve the severity of the disease [22]. The real-time alerts are generated based on parameters, i.e., patients’ clinical visits, travel history, and symptoms identified. The database maintains patients’ travel history, flight origin, health symptoms, etc. To prevent the spread of the Coronavirus, fast COVID-19 tests and clinical assessments are vital for effective decision-making and logistical planning in healthcare. ML tools predict patients’ survival with 90% precision [23]. 

The hybrid ML methods are based on fuzzy inference systems and multi-layered Perceptron for predicting infection and mortality rates [24]. A Fuzzy classifier [25] was used in the classification of EEG signal classification. The outbreak of African swine fever-like diseases was predicted successfully by authors using the Random Forest ML technique and meteorological data [26]. The study emphasized comparative analysis over time series approaches for predicting influenza outbreaks. The study used influenza-like illness data from websites of healthcare centers in Iran [27]. ML techniques play an important role in identifying fever hotspots and disease outbreak predictions associated with climatic factors in Taiwan [28]. The Artificial Neural Network-based approach is developed for predicting COVID-19 outbreaks at the Gulf of Mexico coast [29]. The authors followed the Data Mining approach for predicting dengue outbreaks in Bangladesh [30] and LogitBoost ensemble classifierier in predicting the dengue outbreak and compared their performances [31]. A similar study for predicting dengue outbreak used a Baysian classifier [32] with resonable accuracy. A study on the weather parameters and predicted the dengue cases for New Delhi used Data Mining techniques [33]. The KNN and SVM DM techniques to forecast diabetic patients and the results obtained by the authors show that SVM achieved more accuracy compared to K-Nearest Neighbor [34]. A study for the prediction of diabetic disease used the Backpropagation algorithm. The study proposed by the authors was implemented in the R programming language [35]. The results generated in the study are compared with J48, SVM, and Naive Bayes. A classification approach has been used on the voice data for comparing different feature sets using a Random Forest classifier using PCA for the dimensionality reduction of feature sets [36]. The study focused on implementing intelligent machine techniques for the real-time forecasting of COVID-19 cases, a model that uses the data from internet searchers, news alerts, and mechanical models for forecasting diseases [37].

Clinical practice guidelines play an important role in reducing medical errors. These guidelines stress the need for resolving ambiguity and vagueness in clinical practice to prevent healthcare failures [38], and reducing the vagueness in medicine can improve clinical decision-making [39]. The authors devised reliability engineering methods to overcome the medical errors. In the proposed study, a reliability analysis was performed on the COVID-19 patients. A new method for the mathematical representation of system development based on uncertain and incompletely data is proposed [40].

Clinical data and a mathematical model for predicting the critical condition of patients suffering from COVID-19 through a smartphone sensor were used [41]. Yan et al. [42] used a similar technique for predicting the survival of patients suffering from COVID-19. The performances of the classification models using Multitree XGBoost algorithms for forecasting COVID-19 infection cases in provinces of South Korea [43] are discussed. A Transfer Learning approach with the Convolution Neural Network (CNN) technique was implemented to detect COVID-19 from X-ray images [44]. This study is a systematic review of COVID-19 using Machine Learning and Deep Learning techniques [45] and a study that has elevated the limitations associated with COVID-19 prediction.

AdaBoost and random forest algorithms are implemented for classifying the regional daily growth rate of the spread of an epidemic. The experiments were performed for 19 regions of Italy to classify the daily COVID-19 growth rate factor based on environmental factors and containment measures [46]. Three Machine Learning models, i.e., Convolutional Neural Network (CNN), DTree Classifier, and BayesNet, along with the environmental factors, were used to classify the COVID-19 cases. In addition, the authors conducted a study to identify the best classification model to classify COVID-19 by using significant weather features chosen by the Principal Component Analysis (PCA) feature selection method [47]. The study performed a predicted severity of COVID-19-infected patients using classification models like the Artificial Neural Network (ANN), SVM, and Random Forest [48]. The authors mechanized the Deep Learning-based model for predicting the mortality rates in COVID-19 patients [49]. Deep Learning techniques can manage and analyze vast biomedical image datasets, which is helpful in real-time applications. A Deep Learning-based fusion model can be used for colorectal cancer disease diagnosis and classification [50]. Predicting the in-hospital mortality caused by COVID-19 is being analyzed using an Ensemble-based Deep Neural Network [51]. The study has developed a COVID-19 mortality tool using the XGBoost ML technique for hospitalized COVID-19 cases [52]. The authors of a study on predicting mortality cases in South Korea using a classification technique used the LR, SVM, KNN, Random Forest, and Gradient Boosting methods for predicting the mortality cases [53]. Techniques like Fuzzy and Deep Learning are important in improving the quality of images related to the healthcare sector. The work was based on Fuzzy Multilevel Image thresholding using an improved Coyote Optimization Algorithm [54]. The techniques like deep reinforcement learning are employed in areas like anomaly detection that combine reinforcement learning and deep learning, which enables artificial agents to learn knowledge and experience actual data directly [55]. Further, big data analytical techniques are becoming ubiquitous for achieving optimized results and improving classification performances [56]. The works and techniques followed by the different authors in the related works are summarized in Table 1.

The proposed studies have focused on ensemble classifiers for forecasting disease outbreaks in the provinces of India. The proposed Fine-tuned Ensemble classifier is compared with the state-of-the-art models concerning prediction accuracy. The AI, ML, and Big Data techniques are implemented to forecast COVID-19. In the proposed study, predictions are performed for forecasting the death rates of infection and patients cured of the virus. The performance evaluation of the classification algorithms, which include SVM, Naïve Bayes, Decision Tree, and Ensembling methods on the recent dataset related to COVID-19, is done to the proposed Hybrid model. The accuracy and other performance measures of the proposed Fine-tuned Ensemble model are better than the existing Ensemble Classification model. In the related works, the performances of various existing classifiers are presented. The proposed model has outperformed existing models in terms of accuracy and precision.

## 3. Machine Learning for Predictive Analytics

ML techniques play an important role in different sectors of society to discover the latest trends and perform predictive analytics. Still, there are a lot of challenges to be faced. The classification techniques require a dataset with a large volume for better precision, which is a challenging task for unknown diseases. The datasets in different areas are available on internet resources, but finding the appropriate dataset for performing experiments is challenging. The attributes and data types taken in the dataset also vary as per the individual’s requirements; therefore, attaining similarity in the dataset structure is one of the significant challenges. In such cases, the dataset sometimes needs to be synthesized or semi-synthesized. Another major challenge is the quality of the dataset. Many of the datasets contain missing values, i.e., outliers, resulting in lower performances of the classification algorithms in terms of accuracy. In such a scenario, the dataset is to be preprocessed to remove the outliers to boost up the performances of classification algorithms.

The classification algorithms suffer from underfitting and overfitting problems, leading to poor performance and a lot of variance in the performing predictions, and the results are biased with poor outcomes. The underfit model performs with few features and without understanding the training dataset, leading to overfitting. The predictive analyses of classification performed on the test data to check the accuracy of the model’s accuracy or detect overfitting are: (a) cross-validation, (b) early stopping, (c) pruning, (d) ensembling, (e) voluminous training sample, (f) removing unwanted attributes, etc.

**Figure 1:**
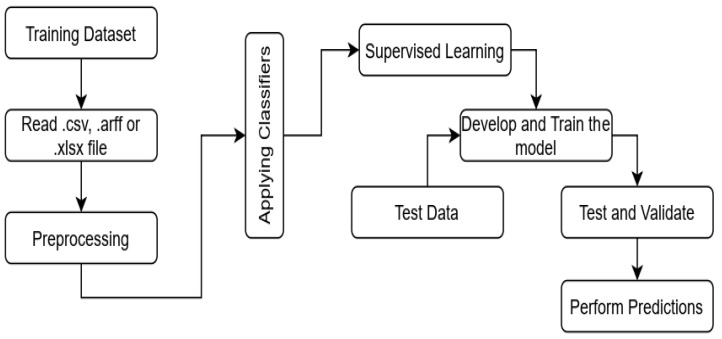
Workflow of classification models for predictive analytics.

The generic workflow of classification models for performing predictions is shown in Figure 1. The workflow in Figure 1 is shown with all three Machine Learning techniques, but only classification techniques are implemented in the proposed work. The ML algorithms for data classification are implemented, and the dataset over which the model is trained for predictions consists of labeled input with a well-defined target class. The dataset in the form of .csv, .arff, or .xlsx files is read in the first step. The dataset is preprocessed to clean up the data and remove the outliers. The missing values are removed and replaced by calculating the mean of the values to enhance the performance of the classification algorithms. Generally, the classification techniques are performed after preprocessing of the dataset.

The Supervised Learning models rely on the training set for precise data classification. The Supervised Learning technique outperforms in dealing with classification problems and regression tasks, and there exists a conception of the output along the learning process. The training dataset taken for experimentation in the proposed study is labeled and requires a training process that is one of the Supervised Learning requirements. The Fine-tuned Ensemble technique allows evaluating and reusing the results for a new sample, which helps validate the results. In our experiment, to maximize the ML model’s performance and acquire a good predictive performance, overfitting and the underfitting problem of the model are removed [57]. The dataset is split into training and testing data so that the ML model performs well on training data rather than on hidden and unseen data. The test data is passed into the trained model for testing, validation, and final performance. ML tools and techniques are widely used in performing predictions. ML techniques have benefitted the health industry in personalized healthcare, electronic healthcare record maintenance, technology-enabled diagnostics, infectious diseases predictions, etc. [58].

### 3.1. Ordinal Decision Tree

An ordinal classification is a significant group of real-time challenges that involve ordering both the properties of a structure to be categorized and the groups. This ensures that decision trees are adaptable prediction models that do not need additional parameters when new features are added. They may yield either a definite outcome from the input data or the numerical prediction outcome. The decision trees consist of the nodes and the branches. At every instance, one of the features is assessed to decide the data during the training process, and predictions are made accordingly. The decision tree classifies and calculates the probability of a given input feature on the most likely category class. The decision tree predicts the target class values, inferring the if-then rules from the input features. The decision tree classifier calculates each attribute’s entropy and information gain and selects the highest attribute. It splits the other input features of the training set and comes relatively closer to the decision. The entropy calculation is an important factor for building the decision tree. The equation for finding the entropy for building the decision tree is shown in Equation (1), and the equation for finding the information gain metric is shown in Equation (2).

(1) $$E(S)(\mathrm{Attrib})=\sum_{j=1}^{n}-p_{j}\log_{2}p_{j}$$

Here, E(S) is the entropy of the attribute for sample set S, and *pj* is the probability of an input feature.

(2) $$\mathrm{IG}\left(S,{\mathrm{Attrib}}_{i}\right)=E\left(S\right)-\underset{v\in \mathrm{Values}\left({\mathrm{Attrib}}_{i}\right)}{\sum }P\left({\mathrm{Attrib}}_{i}=v\right)E\left(S_{v}\right)$$

The information gain (IG) calculated for a particular attribute (Attrib_i_) gives knowledge about the target function, given the value of that attribute, i.e., conditional entropy.

### 3.2. Gaussian Naïve Bayes

Naive Bayes is a supervised classification technique for categorizing the data items through the logic of the Bayes Theorem that states that data items that belong to the same category will have identical characteristics. This implies that each pair of data items classified is independent of the others, where a “Gaussian NB Classifier” is used to train the Naive Bayes model. Gaussian Naive Bayes is based on the assumption that each class has a Gaussian distribution. There are a few situations where the data elements in the hyperplane may not interact with each other. In particular situations, Naive Bayes seems to be performing well, and it is conditionally independent.

Gaussian Naive Bayes is evaluated by using the standard deviation of the input variables ‘i’ for every class value. The mean of the data items ‘m(i)’ with ‘x’ instances and the variable ‘i’ is associated with training data with labels.

(3) $$\;m\left(i\right)=\frac{1}{\left(x\times sum\left(i\right)\right)}$$

The standard deviation for every input instance for each class is approximated through root over the mean square variance of each instance ‘i’ from the mean of the data value of ‘i’; x represents the instance, and ‘vi’ represents the ith instance of the variable ‘k’ in the input data. The standard deviation is determined through Equation (4).

(4) $$s_{d\left(i\right)}=\sqrt{\left(\frac{1}{n}\right)*sum\left(k_{i}-m{\left(i\right)}^{2}\right)}$$

Gaussian Naive Bayes relies on the Gaussian Probability Density function, which approximates the probability of the new value ‘i’, which determines the belongingness of the input values in association with the pretrained data of the model. The probability density function is approximated through Equation (5).

(5) gpdf(i,m(i), s_d(i))={1(sqrt(2×c)×s_d(i)) }×{exp(−((i−(m(i))2/(2×s_d(i)2))))} 

where variable ‘i’ is the input for the density function, and variable ‘c’ is the numerical constant determining the range of the approximated value. The value is approximated in connection to the assumed real-time problem. The function exp() is concerning to the Euler’s number. The approximated value determines the ratio of the patients that might result in death or cures from COVID-19 from the approximated dataset.

### 3.3. Support Vector Machines

The Support Vector Machine (SVM) is an information classification technique used in predictive processing that categorizes new data items according to a predefined set of labeled classes. SVM is a binary classifier; it assumes that the input data contain two possible target values. The supervised classification analyzes 19 affected individuals concerning the patients’ death and cured rates. The SVM framework is a collection of machines, implying that the objective function handles a regression query. While working with a nonlinear regression problem, the input variable x is transformed into a high-dimensional feature space using a nonlinear activation function. Then, linear regression is performed on the space.

SVM models are trained by optimizing the width of the margin that defines the distance between the decision boundary and the closest instance. Moreover, when the assumed problem is not differentiable, there is a real decision limit, and therefore, no “hard-margin” assessment is possible. The objective function for the same is assessed through Equation (6):

(6) $$Obj_{fun}=\frac{1}{2}\times w^{t}\times w+k\times \sum \varepsilon_{i}$$

w—It is the weight associated with the concerning feature dimension.

k—A problem-specific value that determines the margin’s softness-specific assumed context.

$\varepsilon_{i}$—The slack vector indicates the extent of inclination towards the off-target in the considered training data.

The optimal predictive SVM model recommends that the larger value associated with the weight ‘w’ that is not associated with the variable w is completely ignored. The value of ‘k’ is assumed to be a non-zero value that will yield a better resultant outcome. The slack vector is crucial in demining the optimality of the model. When the value is zero or very close to zero, it is assumed that the model is making a precise prediction, and a value beyond one is considered as a wrong prediction. The variables ‘w’ associated with the weight and the bias ‘b’ in the decision hyperplane that assist in differentiating death from curing of the COVID-19 patients is approximated through Equation (7) for the new data variable ‘x’.

(7) $$f\left(x\right)=w^{T}\times x+b$$

The variable ‘f(x)’ determines the distance among the variables in the hyperplane, and the variable ‘b’ is the context-dependent bias value. The approximated value is used to predict the new data of the model.

### 3.4. AdaBoost Algorithm

AdaBoost, introduced by Schapire and Freund [59], is an approach based on Machine Learning, also known as Adaptive Boosting, and it combines the weak classifiers and inaccurate rules into a strong one. The outcome of the weak learners is integrated into a weighted sum. In Adaboost, a weak leaner is the learner with less than 50% error over any distribution, and the Strong classifier is the thresholded linear combination of the weak learner outputs. A classifier with 50% accuracy is given zero weight, and a classifier with less than 50% accuracy is given negative weight. In the algorithm [60,61], the training set with two classes is defined as S = {($x_{1},{\;y}_{1}),\ldots \ldots \ldots ,x_{n},{\;y}_{n})$}. The weights are initialized for the training examples $D_{i}^{1\;}=\frac{1}{N}\;\mathrm{for}\;i=1,2,\ldots \ldots ,N$. The misclassification error of the classifiers fits the training data, using weight w_i_ = 1 to m weak classifiers, as computed in Equation (8):

(8) $$\;\in_{j}=\sum_{i:h_{j}\left(x_{i}\right)\neq y_{i}}D_{i\;}^{j}$$

The final predictions are performed using Equation (9), where (x) is the output of weak classifier j for input x, and H(x) is the final classifier. Here, $\propto_{j}$ is the weight assigned to the jth classifier and is the confidence of the jth model, as shown in Equation (10).

(9) $$H\left(x\right)=\mathrm{sign}\left(\overset{m}{\underset{j=1}{\sum }}\propto_{j}h_{\begin{array}{c} j \end{array}}\;\left(x\right)\right)$$

(10) $$\propto_{j}=\frac{1}{2}\log\left(\frac{1-\in_{j}}{\in_{j}}\right)$$

$\in_{j}$ is the weighted error of the jth model calculated for j = 1 to n iterations. A hypothesis is obtained for the classifier $h_{j}\;$that minimizes $\in_{j}$ and satisfies the condition $\in_{j\;}\leq 1/2$.

The weights of the training examples are updated for the next iteration, as shown in Equations (11) and (12). The weights are normalized by normalizing each of them by the sum of all the weights. The normalized form is shown in Equation (13).

(11) $$D_{i}^{j+1}={\;e}^{-y_{i}h_{j}\left(x_{i}\right)\propto_{j\;}}D_{i}^{j\;}$$

In Equation (4),${\;D}_{i}^{j\;}$ is the weight at the previous level.

(12) $${\;D}_{i}^{j+1}=\frac{D_{i}^{j+1}}{\sum_{i=1}^{n}D_{i}^{j+1}}$$

(13) $$\overset{n}{\underset{i=1}{\sum }}D_{i}^{j+1}=1$$

### 3.5. Random Forest and Bagging

Random Forest, proposed by Ho [62], is an ensembling method and performs classification and regression tasks on decision trees during the run time. Random forest gives an output in the form of classification and generates the average prediction of individual trees, i.e., performs a regression task [63]. The advantage of this method is that it overcomes the problem of overfitting, as seen in the case of decision trees [64].

The bagging algorithm proposed by Leo Breiman [65] is also known as bagging predictors or Bootstrap Aggregating, and it calculates the aggregate of divergent versions of a predicted model. Each such model is trained separately, and then, the results are pooled by averaging. A regression or classification method is performed for each bootstrapped sample once the samples are initially generated. As shown in Equation (14) [66], an average is computed from all of the anticipated outputs for regression. The soft voting method, in contrast, selects the most likely class by using the class’s most likely probability as the output or aggregate. Bagging reduces the variance and avoids overfitting problems. Given a training set for two classes, S = {($x_{1},\;y_{1}),\ldots \ldots \ldots ,x_{n},\;y_{n})$}. A machine is trained on each Si, i = 1 ….to T samples and obtains a sequence of T outputs f_1_(x)……..f_T_(x).

(14) $${\bar{f}}_{bag}={\bar{f}}_{1}\left(x\right)+{\bar{f}}_{2}\left(x\right)+\ldots +{\bar{f}}_{b}\left(x\right)$$

Here, ${\bar{f}}_{bag}$ is the bagged prediction, and ${\bar{f}}_{1}\left(x\right)$……..+${\bar{f}}_{b}\left(x\right)$ are the individual learners.

The final aggregate classifier for regression is shown in Equation (15):

(15) $${\bar{f}}_{x}=\sum_{i=1}^{T}f_{i}\;\left(x\right)$$

Here, x is the point and the average of ${\bar{f}}_{i}$ for i = 1…….T.

The final aggregate classifier for classification is shown in Equation (16):

(16) $$f\left(x\right)=sign(\;\sum_{i=1}^{T}f_{i}\;\left(x\right))$$

## 4. Proposed Fine-Tuned Ensemble Classifiers

**Figure 2:**
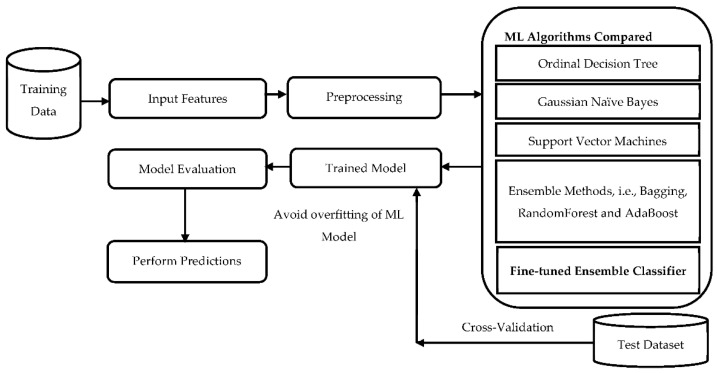
Workflow of the Fine-tuned Ensemble Classification model.

In the proposed work, ML classifiers, i.e., Decision Trees, Naïve Bayes, Support Vector Machines, and Ensembling methods, are implemented in the dataset shown in Table 2. The metrics of the ML algorithms are compared. The experimental results are performed in MATLAB R2021 and the Weka 3.8.5 simulation tool to predict the COVID-19 infection status in different states in India. The ML prominent classifiers taken in the proposed work are also evaluated by their performances. The objective of the experiment is to predict the COVID-19 infection status, i.e., patient death rate, patients cured of COVID-19, and the confirmed cases in different states. The data considered for the predictions is recent, i.e., January–May 2021. The concern of the suggested method is to perform the predictive classification of the spread of the virus in different states. For this purpose, the dataset is obtained from the internet. The current section is organized as preprocessing and feature extraction in Section 4.1, followed by Section 4.2 with the proposed ensemble classified, and Section 4.3 with the input dataset attributes and other experimental details. The methodology adopted in the proposed work is shown in Figure 2. The dataset is fetched and then preprocessed to remove the missing values in the proposed work. After preprocessing, the ML algorithms as shown in Figure 2 are applied and compared to the dataset to obtain the trained model. After getting the models trained on the dataset, the model’s performance evaluation is done, and predictions are performed.

### 4.1. Preprocessing and Feature Extraction

**Figure 3:**
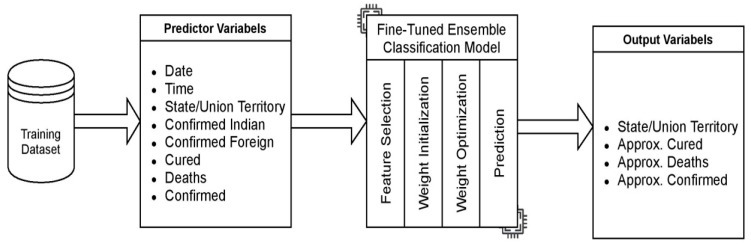
Features selected for the Predictor and Outcome variables.

The dataset is in a structured format and is preprocessed after removing the outliers, i.e., the missing values are removed to improve the ML algorithm’s performance. The input features are extracted and selected as predictors and the outcome variable. The features taken as predictors and the outcome variable for classification are shown in Figure 3. In the proposed work, Neighborhood Component Analysis (NCA) feature selection is implemented in the MatLab environment for classification, and the supported data type is continuous features. Apart from this, the Principal Component Analysis feature method is used to reduce the dimensionality of the dataset without the loss of significant information. The Predictor attributes contain numeric and categorical values. In Supervised learning, the input label is well-defined, i.e., the observed data and the variable to be predicted, i.e., the target variable. Here, the machine is trained using well-labeled data, and the inputs and outputs are matched.

### 4.2. Class Incorporation for the Ensemble Model

It has been observed from the results that the ensembling classifiers, i.e., Bagging, AdaBoost, and Random Forest, have incorrectly classified the instances and have performed poorly compared to Decision Trees, Naïve Bayes, and SVM. The accuracy achieved by Ensembling classifiers is meager compared to them. Therefore, a Hybrid method is proposed to boost the performance of the Ensembling classifiers. The proposed Hybrid model attained the highest accuracy of 94%, and the F-Measure and Recall values of the Hybrid model are higher than the other classifiers. The accuracy and computation time by different ML models are shown in Table 3.

In the proposed method, a ‘vote’ class is implemented in Weka 3.8.5 to combine the probability distributions of these base learners, i.e., AdaBoost, Bagging, and Random Forest, to boost the performance of the weak learners and increase the weight of weak learners who were misclassified in the previous iteration and training model in a dataset. In the proposed approach, these classifiers are considered slow learners, and they are combined to classify the instances correctly. In the proposed Hybrid ML model, the first step is to select the AdaBoost classifier, and the DecisionStump tree class is selected. Then, in the Bagging classifier, a fast decision tree learner (REPTree) is chosen. A decision/regression tree is created with information gain/variance over ten rounds and pruned using the reduced error approach with backfitting. The Random Forest classifier is used after Bagging for constructing a forest of random trees.

In the AdaBoost classifier, the JRip class is implemented. This class implements a propositional rule learner, repeated incremental pruning to produce error reduction (RIPPER) proposed by Cohen [67]. In this algorithm, the building and optimization stages are seen. In the Building stage, it follows the Growing phase and Pruning phase. In the Growing phase, the greedy approach is used. The method does a brute force search across every attribute’s potential value and chooses the condition with the maximum information gain, as shown in Equation (2). In the Pruning phase, incremental pruning of each rule is done, allowing the pruning of any final sequences of the antecedents. In the optimization stage, after generating the initial ruleset {Ri}, two variants of each rule Ri are generated and pruned from randomized data using the procedure followed in the Building stage. Still, one variant is developed from an empty rule while the other greedily adds antecedents to the original rule.

The minimum DL for each variation and the original rule is calculated. The preferred variant has the minimum DL in the ruleset to choose the most appropriate Ri variation. When all of the rules in {Ri} have been considered, and if there are still any positives left behind, the Building stage is again used to create new rules based on those residual positives. When you finish the final step, you should remove the ruleset’s rules, which will raise the ruleset’s overall DL.

### 4.3. Dataset and Implementatiomn Framework

The dataset for the proposed work is obtained from the Kaggle website and is available on the internet [68]. The dataset is recent and contains the years 2020 and 2021 until May 2021, but the dataset from January 2021 to May 2021 is considered for the proposed work. The dataset consists of nine features: Serial number, Date, Time, State/Union Territory, Confirmed Indian National, Confirmed Foreign National, Cured, and Deaths. The experimentation is performed in MATLAB version R2021 with Machine Learning Toolbox and Weka 3.8.5 simulation tool. The machine used in the current study has an Intel(R) Core(TM) i3-7100 CPU @ 3.90GHz, and the Operating System is Windows 10 Pro. The total number of instances in the dataset is 5004, and the number of features is 9. The ML models trained over the dataset in the proposed work are ‘Decision Tree’, ‘Naïve Bayes’, ‘Support Vector Machines’, and ‘Ensembles’. The ‘state/union territory’ is taken as the outcome variable, and the other attributes are predictors. In the proposed work, the attributes ‘Cured’, ‘Deaths’, and ‘Confirmed’ cases are taken as the Predictors for displaying the death rate and patients cured rate from COVID-19 across the different states. The correlated task related to the dataset is classification. The data type of the attributes is a combination of Nominal and Categorical. The sample instances of the COVID-19 dataset are shown in Table 2.

## 5. Results and Discussions

The classification algorithms featuring Decision Trees, GaussianNB, Support Vector Machines, and Ensembling methods, i.e., Bagging, Random Forest, and AdaBoost, are implemented to classify data and perform predictions. The performance of the proposed model is evaluated through various metrics like Precision, Recall, F-measure, and Receiver Operating Characteristics, in-line with the other existing studies [69,70]. The classifier algorithms are applied to the dataset to train the predictive model. The performance evaluation of the Supervised Classifiers for the different input features on the training dataset is compared and shown in Table 3. A 10-fold cross-validation technique is applied to a dataset to evaluate the predictive model. The sample dataset shown in Table 2 is split into 80:20, i.e., 80% of the training set to train the model and 20% is a test set to evaluate it. The results obtained from the classification models show the statewise predicted results of the patients ‘death rate’ vs. ‘cured rate’ and the ‘cured’ vs. ‘confirmed’ cases of patients suffering from COVID-19 from January 2021 to May 2021, i.e., in five months. In the proposed study, the impact of vaccination is not discussed. The authors conducted empirical research and did the classification using Machine Learning techniques. In the study, the fine-tuned ensemble model is proposed for predicting the Death rate and Cured Rate from COVID-19 compared with the other classification models. The prediction of the death rate and cured rate of patients statewise helps the administration and local authorities to take preventive measures and work upon the availability and strengthening of the infrastructure in hospitals to prevent people from the impact of the predicted the third wave due to COVID-19.

The performances of various classification models concern the mortality rate prediction of COVID-19 patients using different Machine Learning approaches on different datasets. The current study has compared the results and performances of the ML techniques implemented by predicting the death rates with the proposed fine-tuned ensemble model. The comparative results concerning various evaluation metrics are shown in Table 3.

It can be observed from the above table that the proposed fine-tuned ensemble classifier outperformed the others in precisely identifying the COVID-19 cases with exceptionally high correctly classified instances, The proposed model exhibited a better performance over all the evaluation metrics like the mean absolute error, root mean square error, relative absolute error, and accuracy of the classification in a reasonable execution time. The performances of various existing models for COVID-19 prediction experimented over the data of various countries are presented in Table 4.

**Figure 4:**
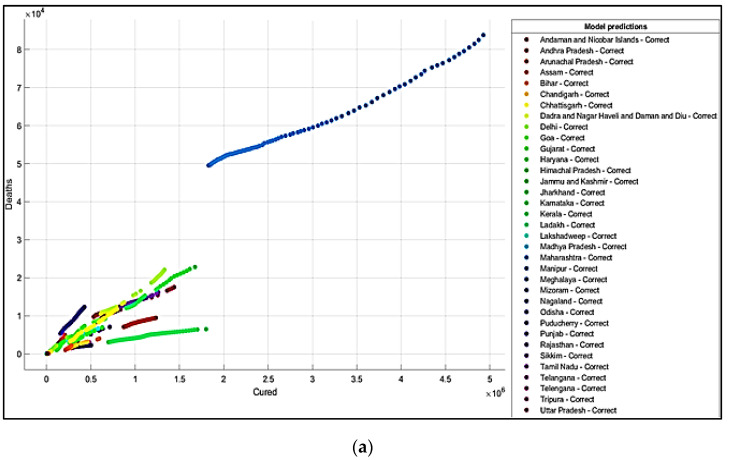

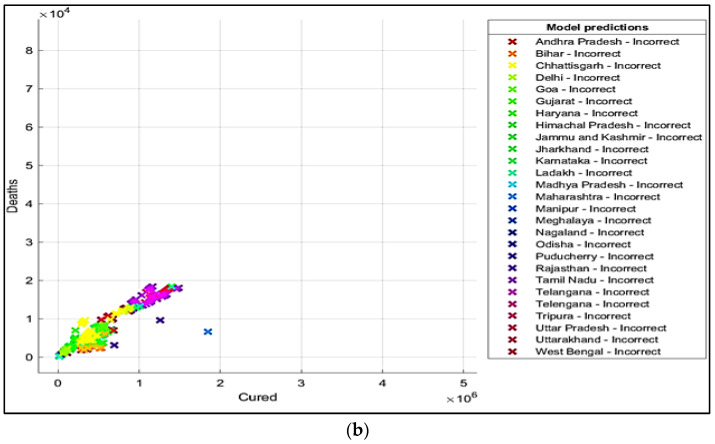
(**a**) Correct Predictions by Decision Tree ‘Cured’ vs. ‘Deaths’. (**b**) Incorrect Prediction by Decision Tree ‘Cured’ vs. ‘Death’.

**Figure 5:**
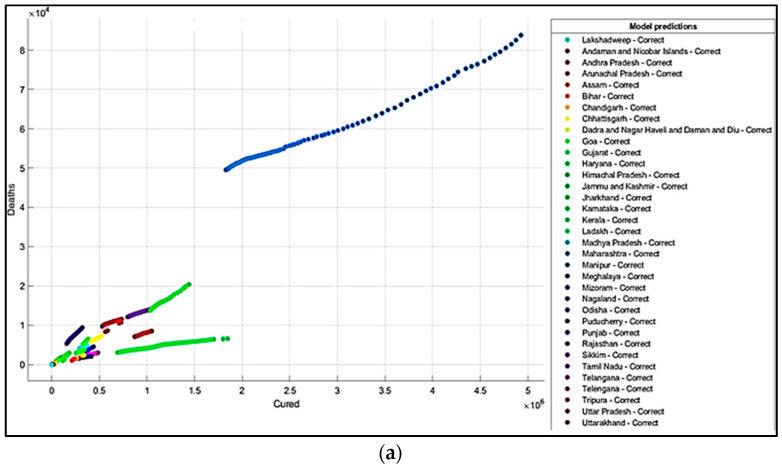

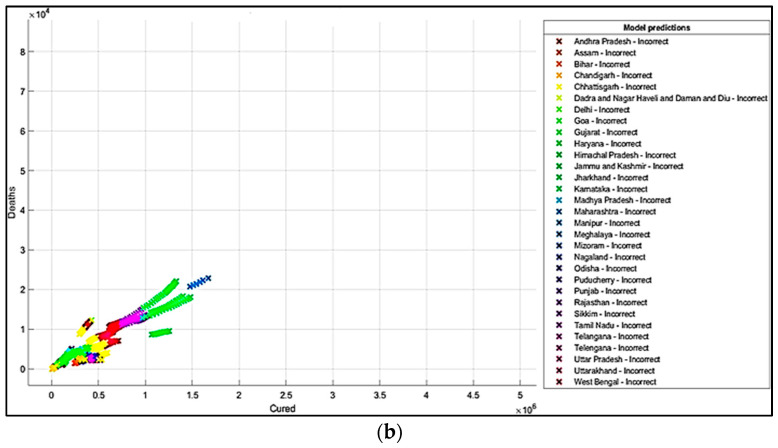
(**a**) Correct Predictions by Gaussian Naïve Bayes ‘Cured’ vs. ‘Deaths’. (**b**) Incorrect Predictions by Gaussian Naïve Bayes ‘Cured’ vs. ‘Deaths’.

**Figure 6:**
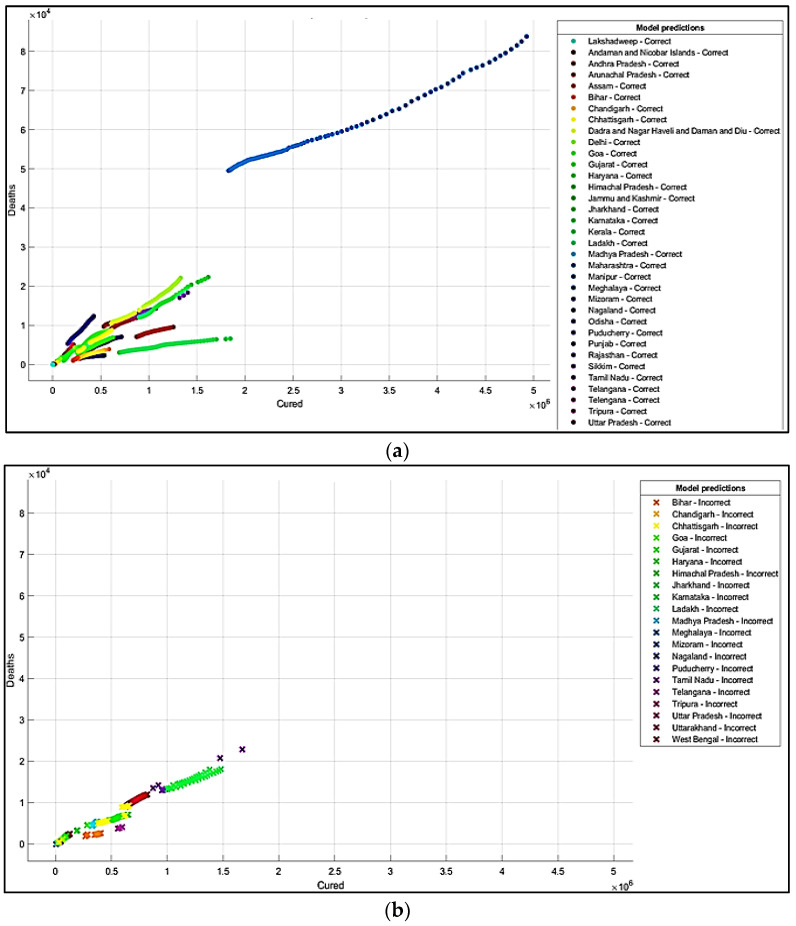
(**a**) Correct Predictions by SVM Model ‘Cured’ vs. ‘Deaths’. (**b**) Incorrect Predictions by SVM Model ‘Cured’ vs. ‘Deaths.

**Figure 7:**
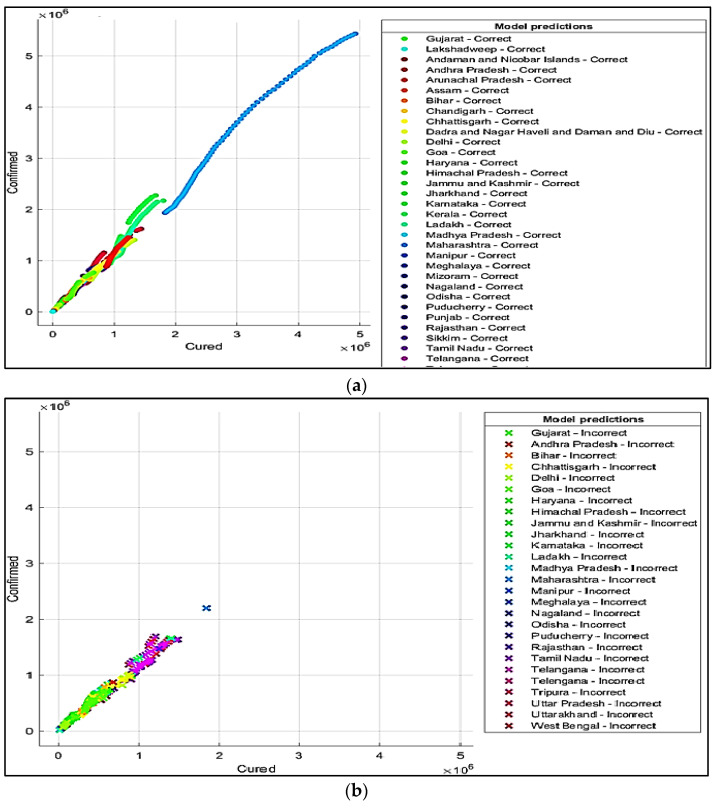
(**a**) Correct Predictions by Decision Tree ‘Cured’ vs. ‘Confirmed’. (**b**) Incorrect Predictions by Decision Tree ‘Cured’ vs. ‘Confirmed’.

**Figure 8:**
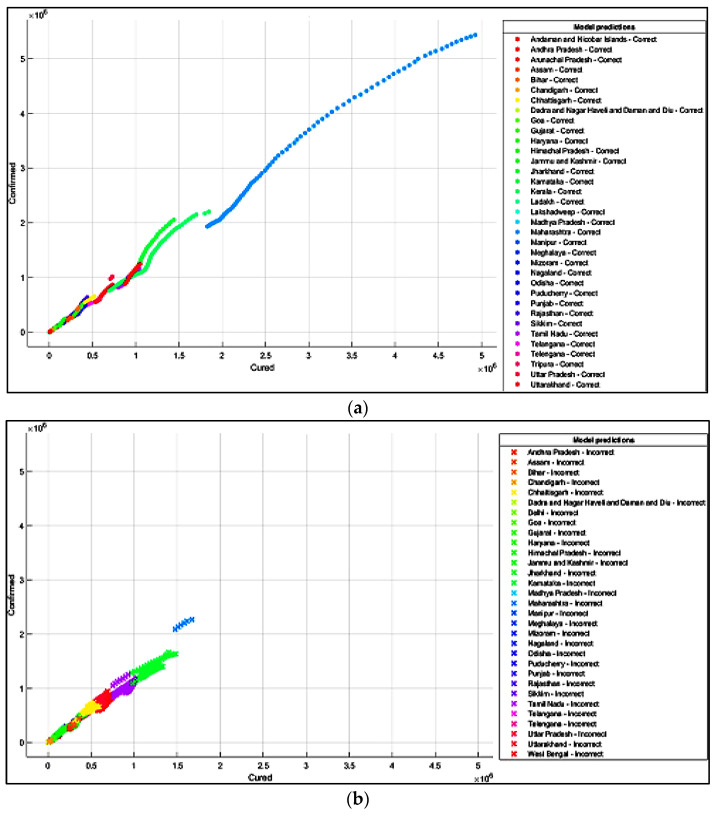
(**a**) Correct Predictions by Gaussian Naïve Model ‘Cured’ vs. ‘Confirmed’. (**b**) Incorrect Predictions by Gaussian Naïve Model ‘Cured’ vs. ‘Confirmed’.

**Figure 9:**
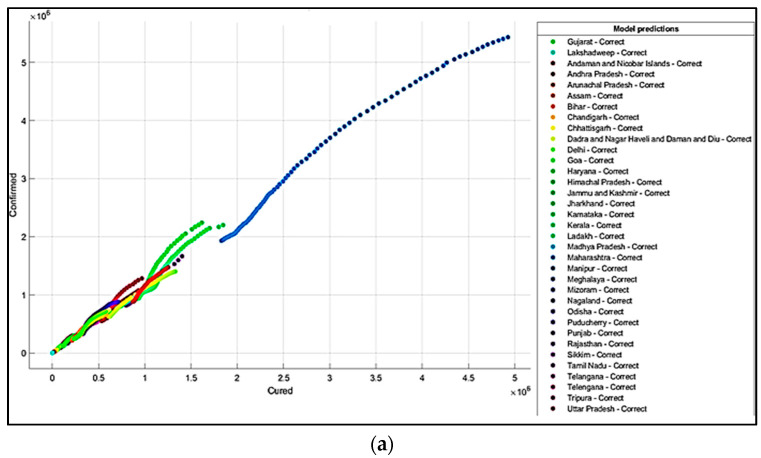

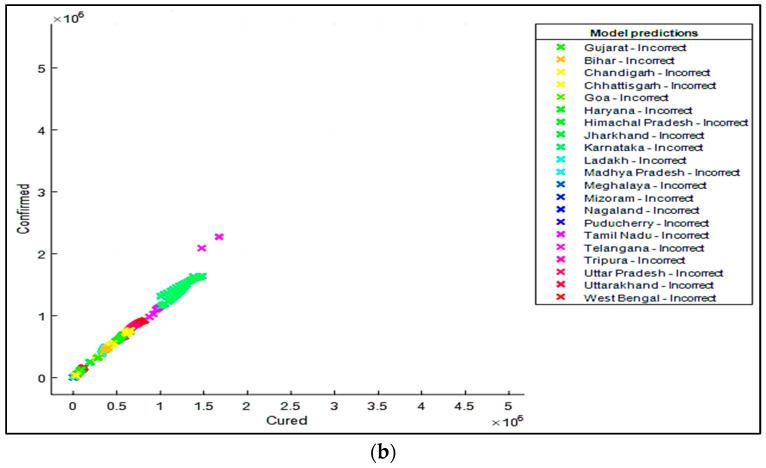
(**a**) Correct Predictions by SVM Model ‘Cured’ vs. ‘Confirmed’. (**b**) Incorrect Predictions by SVM Model ‘Cured’ vs. ‘Confirmed’.

It is summarized from the predicted results that all the ML models have shown Maharashtra, Kerala, Karnataka, Uttar Pradesh, Andhra Pradesh, Delhi, and West Bengal states with more COVID-19 cases compared to the other states. The predicted results in the total number of deaths in Maharashtra is 83,777, and patients cured of the virus are 4,927,480. In Kerala, patients’ death rate value is 6515. In Karnataka, the predicted cured patients are 1,674,487, and deaths are 22,838. After Karnataka, the next state is Uttar Pradesh, where the predicted cured patients are 1,439,096, the death rate values are 17,546, and the confirmed cases are 1,619,645. In a row, the next states having the highest infection cases are Andhra Pradesh and Delhi. The confirmed cases in Andhra Pradesh are 1,454,052, patients cured of the virus are 1,233,017, and the death rate value is 9481. The total confirmed cases in Delhi are 1,402,873, patients cured of infection are 1,329,899, and the total deaths predicted are 22,111, which is comparatively higher than the other states, except for Maharashtra and Karnataka. The correct and incorrect predictive results for predictor variables ‘Cured’and ‘Death’ are shown mainly by Decision Tree, Naïve Bayes, and SVM ML models in Figure 4a,b, Figure 5a,b and Figure 6a,b. The models correct and incorrect predictive results for predictor variables ‘Cured’ and ‘Confirmed’ are shown by Decision Tree, Naïve Bayes, and SVM ML Models in Figure 7a,b, Figure 8a,b and Figure 9a,b.

The statewise detailed accuracy in the form of the True Positive (TP) rate, the False Positive (FP) rate achieved by various Ensemble Classification models concerning the Fine-tuned Ensemble Classification model implemented by the Weka Simulation tool is shown in Table 5. In contrast, Recall and F-Measure values achieved by the ensemble classification models for each state are shown in Table 6. Recall determines the number of correct forecasts produced by the classification model for the complete positive traits present in the dataset. F-Measure is an evaluation metric for combining recall and precision measures into a single statistic that reflects both metrics while weighting them equally. The performance mentioned above the evaluation metrics is the most dominantly used assessments in determining the optimality of the model. The result shows that the proposed Fine-tuned Ensemble model achieved an accuracy of 94%, which is the best value among the various classifiers. The model’s accuracy is defined as the number of correct predictions performed to the total no. of predictions. The recall value determined by the True Positive Rate of the Model is also equivalent to 94%. The recall value shows the intelligence of the classifier to find all the true samples. The values of TPR and FPR are used in approximating the Recall and F-Measure shown in Equations (17) and (18).

(17) $$\mathrm{Recall}=\frac{True\;Positive\left(tp\right)}{True\;Positive\left(tp\right)+False\;Negative\left(fn\right)}$$

(18) $$F-\mathrm{Measure}=\frac{2*Recall*Precision}{\left(Recall+Precision\right)}$$

**Figure 10:**
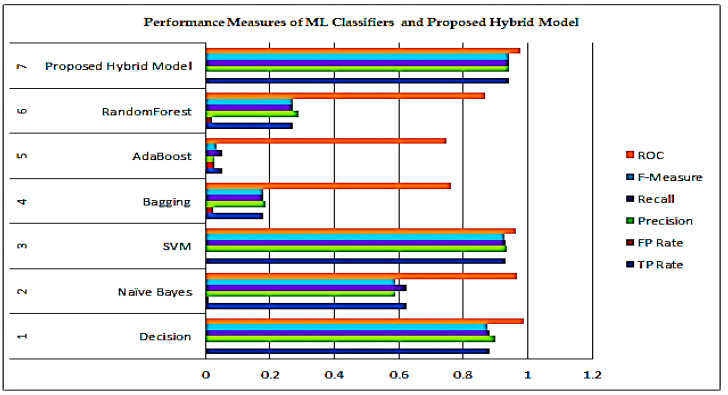
Performance measures of the ML Classifiers and Proposed Hybrid model.

The Receiver Operating Characteristics (ROC) are a metric to evaluate classifier output quality. The ROC curve shows the relationship between sensitivity and specificity of a Machine Learning model. The ROC is a tool with a binary classifier and a plot between True Positive Rate (TPR) values and False Positive Rate (FPR) values. The classification results of the Ensemble Classifiers are better visualized in Figure 10. It is desired to have higher Recall and F-Measure values to model the optimal performance. The TP Rate, FP Rate, Precision, Recall, i.e., the sensitivity of a model, F-Measure, and ROC curve values are shown in the form of a confusion matrix. The confusion matrix of the ML models over the dataset is shown in Table 7. The confusion matrix also summarizes the correct and incorrect predictions of each class. The average True Positive value (TP) of the proposed Fine-tuned Ensemble model is the highest, approximated as 0.94, which means that the Fine-tuned Ensemble model correctly predicts the right class/category, i.e., State/Union Territory. In contrast, False Positive (FP) means the model incorrectly predicts the right class. Although the actual instance was negative, the model projected that instance would be positive. It is desired to have a minimum FP value for better performance. The proposed model has exhibited the highest TR rate among all the models and the least FP rate among the models considered in the model’s statistical analysis.

**Figure 11:**
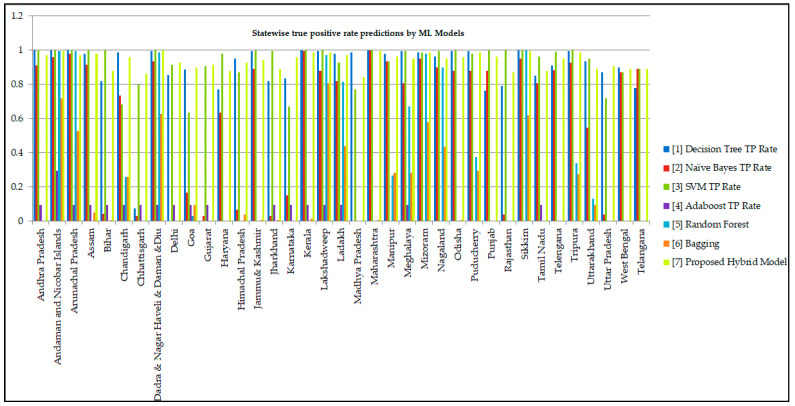
True Positive Rate Predicted by Classification Models for each class.

**Figure 12:**
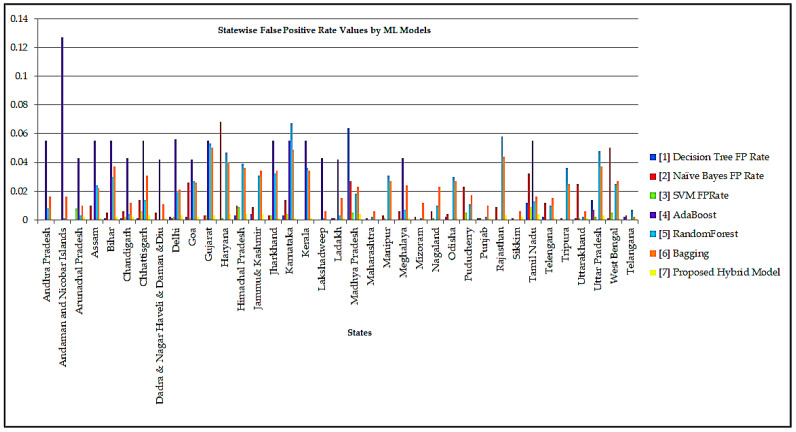
False Positive Rate Predicted by Classification Models for each class.

It can be observed from the experimental results presented in Table 7 that the proposed model has exhibited better performances with better precision over its counterparts. The True Positive rate that depicts the proportion of correct predictions is highest among all the ensemble models considered for evaluation. The True Positive and False Positive predictions of the COVID-19 infection across various Indian states are shown in Figure 11 and Figure 12, respectively.

The proposed Fine-tuned Ensemble Classifier outperforms the conventional classifiers in approximating the number of COVID-19 instances. The model has been evaluated by various metric evaluations. The model has exhibited better performance. The proposed model would assist in better preparedness for future outbreaks and arranging the necessary facilities.

## 6. Conclusions

In the current study, a Fine-tuned Ensemble model for COVID-19 instance classification is done. The predictive analytics using various Ensemble Classification model performances are broadly analyzed through various evaluation metrics. The influence and growth rates of the COVID-19 cases and patients’ recovery rates from the virus in different regions of India are predicted using various classification techniques, which assist in forecasting the impact of COVID-19 cases across the Indian states. The approximated figures would assist the local authorities to strengthen the healthcare infrastructure and preparedness for controlling the spread of the virus, especially in those states where predicted results have shown higher COVID-19 cases. As part of the study, the performances of various ensemble classifiers that include Decision Trees, Gaussian Naïve Bayes, and Support Vector Machines are considered for evaluation, along with the proposed Fine-tuned Ensemble model. The proposed model has outperformed various existing models in assessing COVID-19 cases with better accuracy. The proposed model has needed slightly more execution time than the other models but has exhibited a better accuracy. It is desired to have a Self-Learning for a minimal training and processing latency with better accuracy [78]. The deep learning models can address the issue of imbalanced datasets through high nonlinearity in the classification of instances.

## 7. Future Scope

The predictions are being performed with Machine Learning algorithms for forecasting the growth rate of COVID-19 cases and the recovery rate of patients from the virus in different states. The study is confined to the analysis of COVID-19 impact over the Indian states, and the model can be further evaluated against the datasets of other countries for predicting the cured and death rates. The model’s performance can be analyzed working with other feature sets. It is concluded from the experimental results that the proposed Fine-tuned Ensemble model has achieved the highest accuracy, followed by Support Vector Machines in performing correct predictions of the COVID-19 cases in different states. Machine Learning applications help predict the further spread of the COVID-19 pandemic, extracting significant epidemic trend information associated with the virus. The proposed work can be further enhanced with deep learning-based models for personalized healthcare to patients using the IoT and Machine Learning as the future scope of work. The deep learning framework can be used for more precision in spreading the infection. The models can be developed in the future scope of works to predict respiratory disease infection patterns, virus variants, and peak levels apart from cumulative reports like confirmed, new, and death cases of COVID-19 worldwide. The COVID-19 outburst is controllable if far-reaching and strict, and disciplinary control measures are taken with the collective efforts from society, science, and technology. The current study is limited to the involvement of the epidemiologic expertise in evaluating the performance of the analytical model, and the same might be considered in evaluating future studies.

## Figures and Tables

**Table 1:** The various Machine Learning models in COVID-19 prediction.

Reference	Technique Followed	Work Done
[10]	Decision Tree and Random Forest Algorithms	Classifying ECG signals
[12]	Exploratory Data Analysis	Exploratory data analysis and visualization are performed for virus-infected, recovered, and death cases through classification techniques
[14]	Linear Regression, Multilayer Perceptron, and Vector Auto Regression Methods	Forecast the pandemic
[20]	Digital Signal Processing	Classification of the COVID-19 genomes analysis performed with precision
[21]	AI Framework	A mobile phone-based survey was conducted in provinces that are under quarantine
[22]	Big Data Analytics	The study discussed the response to COVID-19 in Taiwan
[24]	Fuzzy Inference System And Multi-Layered Perceptron	Predicting infection and mortality rates due to COVID-19 for Hungary
[25]	Fuzzy Classifier	EEG Signal Classification is done
[26]	Random Forest	The outbreak of African fever-like diseases was predicted successfully.
[27]	Comparative Evaluation of Time Series Models	Forecasting of influenza diseases outbreak in Iran
[28]	Deep AlexNet Model	Identifying fever hotspots and diseases outbreak predictions associated with climatic factors in Taiwan
[29]	Artificial Neural Network	Predicted oyster norovirus outbreaks along the Gulf of Mexico coast
[30]	Data Mining approach	Predicted dengue outbreaks in Bangladesh
[32]	Bayesian Network	Predicted dengue outbreaks in the Malaysian region
[34]	KNN and SVM techniques	Forecast of diabetic patients
[35]	Backpropagation algorithm implemented in R Programming Language	The study has predicted diabetic diseases. The results generated in the study are compared with J48, SVM, and Naive Bayes
[36]	Random forest classifier	Predicted Parkinson’s disease
[41]	Mathematical Modelling	Predicted the critical condition of patients suffering from COVID-19 in Wuhan
[42]	Support Vector Machine	Predicted the survival of patients suffering from COVID
[43]	XGBoost, Multioutput Regressor	Forecasting COVID-19 infection cases in provinces of South Korea
[44]	Convolution Neural Network (CNN) and Transfer Learning Approach	The technique implemented for detecting the COVID-19 from the X-ray images
[45]	Machine Learning and Deep Learning techniques	A systematic review was conducted in the study to detect COVID-19
[47]	Convolutional Neural Network (CNN), DTree Classifier and BayesNet	A study was conducted to identify the best classification model to classify COVID-19 by using significant weather features chosen by the Principal Component Analysis (PCA) feature selection method
[48]	Artificial Neural Network, SVM, and Random Forest	Predicted the severity of COVID-19-infected patients using ML methods
[49]	Deep Learning (DL)	Deep Learning-based model for predicting the mortality rates in COVID-19 patients
[51]	Ensemble-based Deep Neural Network	Predicted the in-hospital mortality due to COVID-19 using routine blood samples
[52]	XGBoost	XGBoost used as a mortality risk tool for hospitalized COVID-19 cases
[53]	LR, SVM, KNN, Random Forest, Gradient Boosting	Predicted the mortality cases in South Korea using classification techniques

**Table 2:** Sample instances of the COVID-19 dataset.

Sno	Date	Time	State/ Union Territory	Confirmed Indian National	Confirmed Foreign National	Cured	Deaths	Confirmed
1	22 March 2020	6:00 p.m.	Delhi	28	1	5	1	29
2	22 March 2020	6:00 p.m.	Gujarat	18	0	0	1	18
3	22 March 2020	6:00 p.m.	Haryana	7	14	0	0	21
4	8 April 2020	5:00 p.m.	Karnataka	-	-	25	4	175
5	1 August 2020	8:00 a.m.	Assam	-	-	30,357	98	40,269
6	22 March 2020	6:00 p.m.	Punjab	21	0	0	1	21
-	-	-	-	-	-	-	-	-
5004	31 May 2021	8:00 a.m.	Mizoram	-	-	9015	38	12,087

**Table 3:** Performances of various classifiers in COVID-19 prediction.

Classifier	Correctly Classified Instances	Incorrectly Classified Instances	Mean Absolute Error	Root Mean Squared Error	Relative Absolute Error	Root relative Squared Error	Accuracy of Correctly Classified Instances	Time Is Taken to Build the Model (in Seconds)
Decision Trees	4422	582	0.0072	0.0634	13.76%	39.12%	88.37%	0.28
Naïve Bayes	3119	1885	0.0231	0.1191	43.95%	73.45%	62.33%	0.02
SVM	4658	346	0.0037	0.0611	7.11%	37.71%	93.09%	128.61
Bagging	897	4107	0.0465	0.1631	88.55%	100.59%	17.92%	0.47
AdaBoost	262	4742	0.0511	0.1598	97.21%	98.60%	5.23%	0.05
Random Forest	1348	3656	0.0464	0.157	88.26%	96.81%	26.93%	3.59
Proposed Model	4704	300	0.0363	0.1145	69.05%	70.62%	94.00%	1.49

**Table 4:** Comparison of relative studies using ML models with the proposed Fine-tuned Ensemble method.

Reference	Technique	Dataset Size	Country	Results
[47]	XGBoost	3062	USA and Southern Europe	Accuracy: 0.85, NPV: 0.93
[71]	SVM(Linear)	10,237	Korea	Accuracy: 0.91
[72]	LR	2307	Madrid	Sensitivity: 0.81, Specificity: 0.81
[73]	Random Forest	567	-	Accuracy: 0.655
[74]	Multilayer Perceptron	302	Nigeria	Accuracy: 0.85
[75]	Random Forest	341	Itlay	ROC:0.84
[76]	Decision Trees	-	Portugal	Sensitivity: 0.95, Accuracy: 0.9, Specificity: 0.86
[77]	ANN	-	-	Accuracy: 0.89
	Proposed Model	5004	India	Accuracy: 0.94, ROC: 97.8, F-Measure: 0.94

**Table 5:** Detailed Accuracy (TP, FP Rate) for each class Using Machine Learning Classifiers.

TP Rate							FP Rate							Class
Decision Tree	Naïve Byes	SVM	AdaBoost	Random Forest	Bagging	Hybrid Model	Decision Tree	Naïve Byes	SVM	AdaBoost	Random Forest	Bagging	Proposed Model	State/Union Territory
1	0.91	1	0.094	0	0	0.971	0	0	0	0.055	0.008	0.016	0	Andhra Pradesh
1	0.957	1	0.295	0.993	0.719	0.993	0	0	0	0.127	0.001	0.016	0	Andaman and Nicobar Islands
1	0.978	1	0.094	0.993	0.525	0.971	0	0	0.008	0.043	0.003	0.01	0.002	Arunachal Pradesh
0.978	0.914	1	0.094	0	0.05	0.978	0	0.01	0	0.055	0.024	0.022	0.001	Assam
0.82	0.043	1	0.094	0	0.007	0.878	0.001	0.005	0	0.055	0.03	0.037	0.002	Bihar
0.986	0.734	0.683	0.094	0.259	0.259	0.957	0.001	0.006	0.002	0.043	0.004	0.012	0.002	Chandigarh
0.072	0.029	0.799	0.094	0	0	0.863	0.001	0.014	0.006	0.055	0.014	0.031	0.003	Chhattisgarh
0.993	0.935	1	0.094	0.986	0.626	1	0	0.005	0	0.042	0	0.011	0.001	Dadra & Nagar Haveli
0.856	0	0.914	0.094	0	0	0.928	0.002	0.001	0.002	0.056	0.019	0.021	0.003	Delhi
0.885	0.165	0.633	0.094	0.029	0.094	0.899	0.002	0.026	0.001	0.042	0.027	0.026	0.002	Goa
0	0.029	0.906	0.094	0	0	0.914	0	0.003	0.003	0.055	0.053	0.05	0.003	Gujarat
0.77	0.633	0.978	0	0	0	0.878	0	0.068	0.001	0	0.047	0.04	0.004	Haryana
0.95	0.065	0.871	0	0	0.036	0.928	0.003	0.01	0.009	0	0.039	0.036	0.004	Himachal Pradesh
0.993	0.892	1	0	0	0.007	0.942	0.004	0.009	0	0	0.031	0.034	0.004	Jammu& Kashmir
0.82	0.029	0.993	0.094	0	0.007	0.892	0	0.003	0.003	0.055	0.032	0.034	0.001	Jharkhand
0.835	0.151	0.669	0.094	0	0	0.957	0.003	0.014	0.004	0.055	0.067	0.049	0.001	Karnataka
1	0.993	1	0.094	0	0.014	0.986	0	0	0	0.055	0.036	0.034	0.001	Kerala
0.993	0.878	1	0.094	0.971	0.806	0.986	0	0	0	0.043	0.001	0.006	0	Lakshadweep
0.978	0.82	0.928	0.094	0.813	0.439	0.971	0.001	0.001	0	0.042	0.003	0.015	0.001	Ladakh
0.986	0	0.77	0	0	0	0.842	0.064	0.027	0.005	0	0.018	0.023	0.004	Madhya Pradesh
1	1	1	0	0	0.007	0.993	0	0.001	0	0	0.002	0.006	0	Maharashtra
0.978	0.935	0.935	0	0.266	0.281	0.964	0	0.003	0.001	0	0.031	0.027	0	Manipur
0.993	0.806	0.993	0.094	0.669	0.281	0.95	0	0.006	0.001	0.043	0.007	0.024	0.001	Meghalaya
0.986	0.95	0.986	0	0.978	0.576	0.986	0	0.002	0	0	0.001	0.012	0.001	Mizoram
0.964	0.899	0.993	0	0.899	0.432	0.95	0	0.006	0.001	0	0.01	0.023	0	Nagaland
0.993	0.878	1	0	0	0.007	0.957	0.002	0.004	0	0	0.03	0.027	0.001	Odisha
0.993	0.878	0.978	0	0.374	0.295	0.986	0	0.023	0.005	0	0.011	0.017	0	Puducherry
0.763	0.878	1	0	0	0	0.964	0.001	0.001	0	0	0.002	0.01	0.001	Punjab
0.791	0.036	1	0	0	0	0.871	0	0.009	0	0	0.058	0.044	0.003	Rajasthan
1	0.95	1	0	1	0.619	0.993	0	0.001	0	0	0	0.006	0.002	Sikkim
0.849	0.806	0.964	0.094	0	0	0.878	0.012	0.032	0.009	0.055	0.013	0.016	0.004	Tamil Nadu
0.909	0.884	0.992	0	0	0	0.95	0.002	0.012	0	0	0.01	0.015	0.001	Telangana
0.993	0.928	1	0	0.338	0.273	0.986	0	0.001	0	0	0.036	0.025	0	Tripura
0.935	0.547	0.95	0	0.129	0.094	0.892	0.001	0.025	0.001	0	0.002	0.006	0.001	Uttarakhand
0.871	0.036	0.719	0	0	0	0.906	0.014	0.007	0.002	0	0.048	0.037	0.003	Uttar Pradesh
0.899	0.871	0.871	0	0	0	0.892	0.001	0.05	0.005	0	0.025	0.027	0.001	West Bengal
0.778	0.889	0.889	0	0	0	0.889	0.002	0.003	0	0	0.007	0.002	0.001	Telangana
0.88	0.623	0.93	0.052	0.269	0.179	0.94	0.003	0.011	0.002	0.027	0.021	0.023	0.002	Weighted Avg.

**Table 6:** Detailed Accuracy (Recall, F-Measure) for each class using Machine Learning Classifiers.

Recall							F-Measure							Class
Decision Tree	Naïve Byes	SVM	AdaBoost	Random Forest	Bagging	Proposed Hybrid Model	Decision Tree	Naïve Byes	SVM	AdaBoost	Random Forest	Bagging	Proposed Model	State/Union Territory
1	0.914	1	0.094	0	0	0.971	0	0	0	0.062	0	0	0.985	Andhra Pradesh
1	0.957	1	0.295	0.993	0.719	0.993	0	0	0	0.103	0.986	0.631	0.996	Andaman and Nicobar Islands
1	0.978	1	0.094	0.993	0.525	0.971	0	0	0.008	0.072	0.942	0.564	0.954	Arunachal Pradesh
0.978	0.914	1	0.094	0	0.05	0.978	0	0.01	0	0.062	0	0.055	0.968	Assam
0.82	0.043	1	0.094	0	0.007	0.878	0.001	0.005	0	0.062	0	0.006	0.9	Bihar
0.986	0.734	0.683	0.094	0.259	0.259	0.957	0.001	0.006	0.002	0.072	0.369	0.31	0.947	Chandigarh
0.072	0.029	0.799	0.094	0	0	0.863	0.001	0.014	0.006	0.062	0	0	0.879	Chhattisgarh
0.993	0.935	1	0.094	0.986	0.626	1	0	0.005	0	0.073	0.986	0.619	0.989	Dadra & Nagar Haveli
0.856	0	0.914	0.094	0	0	0.928	0.002	0.001	0.002	0.061	0	0	0.912	Delhi
0.885	0.165	0.633	0.094	0.029	0.094	0.899	0.002	0.026	0.001	0.073	0.029	0.093	0.912	Goa
0	0.029	0.906	0.094	0	0	0.914	0	0.003	0.003	0.062	0	0	0.898	Gujarat
0.77	0.633	0.978	0	0	0	0.878	0	0.068	0.001	0	0	0	0.868	Haryana
0.95	0.065	0.871	0	0	0.036	0.928	0.003	0.01	0.009	0	0	0.032	0.899	Himachal Pradesh
0.993	0.892	1	0	0	0.007	0.942	0.004	0.009	0	0	0	0.007	0.91	Jammu& Kashmir
0.82	0.029	0.993	0.094	0	0.007	0.892	0	0.003	0.003	0.062	0	0.007	0.919	Jharkhand
0.835	0.151	0.669	0.094	0	0	0.957	0.003	0.014	0.004	0.062	0	0	0.967	Karnataka
1	0.993	1	0.094	0	0.014	0.986	0	0	0	0.062	0	0.013	0.975	Kerala
0.993	0.878	1	0.094	0.971	0.806	0.986	0	0	0	0.072	0.975	0.797	0.993	Lakshadweep
0.978	0.82	0.928	0.094	0.813	0.439	0.971	0.001	0.001	0	0.073	0.85	0.449	0.964	Ladakh
0.986	0	0.77	0	0	0	0.842	0.064	0.027	0.005	0	0	0	0.848	Madhya Pradesh
1	1	1	0	0	0.007	0.993	0	0.001	0	0	0	0.012	0.993	Maharashtra
0.978	0.935	0.935	0	0.266	0.281	0.964	0	0.003	0.001	0	0.225	0.254	0.975	Manipur
0.993	0.806	0.993	0.094	0.669	0.281	0.95	0	0.006	0.001	0.072	0.705	0.264	0.96	Meghalaya
0.986	0.95	0.986	0	0.978	0.576	0.986	0	0.002	0	0	0.975	0.582	0.982	Mizoram
0.964	0.899	0.993	0	0.899	0.432	0.95	0	0.006	0.001	0	0.804	0.387	0.967	Nagaland
0.993	0.878	1	0	0	0.007	0.957	0.002	0.004	0	0	0	0.007	0.964	Odisha
0.993	0.878	0.978	0	0.374	0.295	0.986	0	0.023	0.005	0	0.423	0.313	0.986	Puducherry
0.763	0.878	1	0	0	0	0.964	0.001	0.001	0	0	0	0	0.961	Punjab
0.791	0.036	1	0	0	0	0.871	0	0.009	0	0	0	0	0.877	Rajasthan
1	0.95	1	0	1	0.619	0.993	0	0.001	0	0	1	0.683	0.965	Sikkim
0.849	0.806	0.964	0.094	0	0	0.878	0.012	0.032	0.009	0.062	0	0	0.868	Tamil Nadu
0.909	0.884	0.992	0	0	0	0.95	0.002	0.012	0	0	0	0	0.954	Telangana
0.993	0.928	1	0	0.338	0.273	0.986	0	0.001	0	0	0.262	0.256	0.986	Tripura
0.935	0.547	0.95	0	0.129	0.094	0.892	0.001	0.025	0.001	0	0.218	0.144	0.922	Uttarakhand
0.871	0.036	0.719	0	0	0	0.906	0.014	0.007	0.002	0	0	0	0.894	Uttar Pradesh
0.899	0.871	0.871	0	0	0	0.892	0.001	0.05	0.005	0	0	0	0.922	West Bengal
0.778	0.889	0.889	0	0	0	0.889	0.002	0.003	0	0	0	0	0.842	Telangana
0.88	0.623	0.93	0.052	0.269	0.179	0.94	0.003	0.011	0.002	0.034	0.271	0.18	0.94	Weighted Avg.

**Table 7:** Performance analysis of various classifiers over the COVID-19 dataset.

ML Classifier	TP Rate	FP Rate	Precision	Recall	F-Measure	ROC
Decision	0.88	0.003	0.899	0.88	0.876	0.989
Naïve Bayes	0.623	0.011	0.588	0.623	0.587	0.968
SVM	0.93	0.002	0.934	0.93	0.929	0.964
Bagging	0.179	0.023	0.187	0.179	0.180	0.761
AdaBoost	0.052	0.027	0.026	0.052	0.034	0.747
RandomForest	0.269	0.021	0.290	0.269	0.271	0.866
Proposed Model	0.940	0.002	0.941	0.940	0.940	0.978

## Data Availability

Not applicable.

## Abbreviations

ANN	Artificial Neural Network
AI	Artificial Intelligence
AUC	Area under curve
CNN	Convolutional Neural Network
DM	Data Mining
ECG	Electrocardiogram
ES	Expert Systems
FPR	False Positive Rate
FNR	False Negative Rate
GIS	Geographic Information Systems
GNB	Gaussian Naïve Bayes
HDFS	Hadoop Distributed File System
KNN	K Nearest Neighbor
ML	Machine Learning
NCA	Neighborhood Component Analysis
NLP	Natural Language Processing
NaN	Not a Number
NN	Neural Network
PCA	Principal Component Analysis
RF	Random Forest
RNA	Ribonucleic Acid
RT-PCR	Reverse transcription-polymerase chain reaction
ROC	Receiver Operating Characteristics
SARS	Severe acute respiratory syndrome
SVM	Support Vector Machines
TPR	True Positive Rate
WHO	World Health Organization
WEKA	Waikato Environment for Knowledge Analysis
